# The Relationship Between Bone Mineral Density and Cardiovascular Function in Duchenne Muscular Dystrophy: A Retrospective Cohort Study

**DOI:** 10.1371/currents.md.ee7ac0ec8c19a47b114737f9c2714779

**Published:** 2018-03-22

**Authors:** Tara Kervin, Mathula Thangarajh

**Affiliations:** Statistics Collaborative, Inc., Washington, D.C., USA; Department of Neurology, Children's National Health System, George Washington School of Medicine, Washington, D.C., USA

## Abstract

**INTRODUCTION:**

Duchenne muscular dystrophy (DMD) is an X-linked genetic disorder that causes progressive skeletal and cardiac muscle weakness in boys. Cardiac dysfunction is a frequent cause of death in DMD. Glucocorticoids are the standard of care in DMD. The long-term use of oral glucocorticoids in DMD is complicated by poor bone health. Epidemiological studies suggest a biological link between the loss of bone mineral density (BMD) and cardiovascular disease, including coronary artery and cerebrovascular diseases. Whether an association between low BMD and cardiac dysfunction occurs in DMD boys has not yet been studied. The objective of this retrospective cohort study was to examine the relationship between BMD and cardiovascular health in DMD.

**METHODS:**

Retrospective data analyses was performed from de-identified medical records from a tertiary academic medical center. Whole body BMD was measured using dual-energy xray absorptiometry (DEXA) scan and left ventricular ejection fraction (LVEF) was measured using echocardiogram. Linear regression was used to evaluate the relationship between BMD and LVEF.

**RESULTS:**

Data was analyzed from a total of 32 boys with DMD. The mean age at which baseline BMD measurements was obtained of 11±3 (SD) years. The worst LVEF was measured at a mean of 23.7±21.8 (SD) months after the baseline BMD measurement. The final adjusted linear regression of the relationship between baseline BMD z-score and worst LVEF was not statistically significant (ß=0.41, p‑value=0.6455).

**DISCUSSION:**

In this cohort of boys with DMD, BMD was not associated with LVEF dysfunction up to 79 months later. Future research with a longer longitudinal follow-up period is warranted to evaluate the relationship between BMD and cardiovascular disease in DMD.

## Introduction

Duchenne muscular dystrophy (DMD) is an X-linked genetic disorder that is caused by mutations in the dystrophin gene, and affects approximately 1 in 3,500-5,000 males.^1^ The life expectancy in DMD in developed countries has improved in the last decade with survival extending to mid-30s.^2^ ^3^ Although progressive loss of skeletal muscle strength is a clinical hallmark of DMD, cardiac dysfunction due to cardiac muscle weakness is often the cause of death in DMD.

Bone health is a significant co-morbidity in DMD. Poor bone health in DMD is multifactorial; decrease in load bearing from progressive muscle weakness, long-term glucocorticoid treatment, and poor bone mineral accrual contribute to the well‑established risks of low bone mineral density and bone fractures.^4^ As oral glucocorticoid treatment is often begun between ages 4-7 years in most boys, low bone mineral density (BMD) precedes cardiovascular disease in DMD. The relationship between BMD and cardiovascular health has been researched in non‑DMD adult population; evidence suggests low BMD is associated with cardiovascular disease, including coronary artery and cerebrovascular events. Notably, twins who were over the age of 50 years with cardiovascular disease (defined as heart failure, stroke, ischemic heart disease and peripheral atherosclerosis), showed that they had a higher risk of developing subsequent hip fracture in a Swedish study. Collectively, these studies suggest that there is an overlap in pathophysiological mechanisms that are yet to be completely unraveled.^5^,^6^,^7^

To date, the specific relationship between BMD and cardiovascular health has not been evaluated in DMD. With the increase in life expectancy in DMD, evaluation of risk factors that impact cardiovascular health are critically important. The objective of the present study was to evaluate the relationship between BMD and cardiovascular health. BMD was measured by dual-energy X-ray absorptiometry (DEXA) and left ventricular ejection fraction (LVEF) was assessed by echocardiogram. We hypothesized that there is a positive linear relationship between bone mineral density and LVEF, with parallel decline in both BMD and LVEF in DMD.

## Data collection

We retrospectively analyzed data from de-identified medical records of boys with DMD who were followed at a single, academic institution. The study, waiver of consent, and waiver of signed consent was approved by the the IRB of Children’s National Health System, Washington, D.C. (IRB#5079). DMD patients who had at least one DEXA scan prior to the first measurement of LVEF were included in the study analyses. DMD boys whose LVEF was less than or equal to 55% predicted value, or those on a cardiac protective agent prior to the baseline DEXA were excluded from the study analyses.

We obtained patient characteristics (age, sex, race, height, weight, ambulatory status, and steroid use), Z-scores from lumbar spine DEXA, and LVEF measurements. BMD measures were dichotomized into low BMD and normal BMD. Low BMD was defined as whole body Z-score of -2.0 or lower, or a physician-confirmed diagnosis of osteoporosis. Missing Z-scores for patients with a physician-confirmed diagnosis of osteoporosis based upon X-ray results were imputed to an arbitrary BMD score of -4.0.

For each subject meeting study inclusion criteria, all available LVEF measurements were first evaluated. The lowest LVEF score after the initial DEXA was chosen. As we aimed to evaluate the relationship between bone mineral density and cardiovascular health outcomes overtime (as measured by change in LVEF) we excluded subjects who already presented signs of LVEF dysfunction at the time of their baseline BMD measurement. LVEF dysfunction was defined as less than or equal to 55% of predicted value. We defined LVEF dysfunction was as less than or equal to 55% of predicted value. A significant and clinically meaningful decrease in LVEF was defined as a 5% change in predicted value based on literature support that a change in LVEF of up to 4% can occur due to random chance.^8^

## Analysis

We used SAS to analyze the data. The following variables were evaluated as potential confounders: baseline age, BMI, ambulatory status, steroid use, and Vitamin D level. We hypothesized that the measure of association between those with and without cardiovascular dysfunction would differ by steroid use; as such, steroid use was assessed for effect modification.

Counts, means, and quartiles were used to summarize the study population. The distribution of the study’s continuous variables was examined for violations of normality and to record missing data. The number of LVEF measurements and the range of time (months) between baseline BMD and the worst LVEF score, as well as the first and last measurements of LVEF, were obtained. With the data being collected retrospectively, there was a lack of standardization in the timing of visits for collection of LVEF measurements. The interval ranged between 1 to 58 months for collection of the first LVEF following the baseline BMD measurement. For sensitivity testing, a “standardized” LVEF measurement was derived by taking the LVEF measurement, if any, within a window of ±3 months of 24 months since baseline BMD measurement. If there was more than one LVEF measurement within this window, the one closest to the mean was chosen for final analysis.

Linear and logistic regression was used to evaluate the association between BMD and LVEF. Identified confounders were included as covariates in the models if found to be statistically significant, or if considered as clinically meaningful. The following secondary analyses of the linear relationship between BMD and LVEF were performed as sensitivity analyses to the primary study findings: (i) linear regression excluding patients with the imputed baseline bone mineral density z-score; (ii) linear regression modeling of LVEF at the standardized visit of ±3 months of 24 months since baseline; (iii) linear regression modeling change in LVEF from first LVEF measurement to the lowest LVEF measurement.

## Results

***Primary results***

The primary analysis included 32 boys with DMD. The mean age was 11±3 (SD) years. Of the 32 boys, 19 had low BMD and 4 had imputed values for baseline BMD. The mean age of those with low BMD was 13±2.7 years, and those with normal BMD was 8.8±2.5 years. The mean BMD z‑score among the 32 patients was -2.2. The lowest LVEF was measured at a mean of 23.7±21.8 (SD) months after baseline BMD measurement (Table 1). The mean age at first LVEF measurement was 12±4 (SD) years. The mean LVEF was 60.1%. There was a total of three LVEF dysfunction events (incidence rate of 9.1 per 100 DMD patients) and four significant decrease LVEF events (incidence rate of 12.5 per 100 DMD patients). The exposed and unexposed groups were concordant in terms of BMI, steroid use, and baseline Vitamin D level.

**Table 1. Patient characteristics and demographics:** 

	Low BMD N=19	Normal BMD N=13	Total N=32
Age (years)			
Mean (SD)	13.0 (2.7)	8.8 (2.5)	11.3 (3.3)
Quartiles (25th, median, 75th)	11.0, 13.0, 15.0	7.0, 9.0, 11.0	9.0, 11.0, 13.5
			
Race, n (%)			
Caucasian	2/6 (33)	2/4 (50)	4/10 (40)
Hispanic	4 (67)	0	4 (40)
Black	0	2 (50)	2 (20)
			
BMI			
Mean (SD)	23.6 (5.2)	22.2 (7.8)	23.0 (6.3)
Quartiles (25th, median, 75th)	20.4, 22.0, 27.3	16.3, 21.0, 22.8	18.5, 22.0, 25.3
			
Using steroids, n (%)	17 (89)	10 (77)	27 (84)
			
Ambulatory, n (%)	5 (26)	2 (15)	7 (22)
			
Vitamin D			
Mean (SD)	24.0 (9.0)	26.8 (5.8)	25.2 (7.8)
Quartiles (25th, median, 75th)	16.0, 24.0, 26.0	25.0, 25.0, 29.0	22.5, 25.0, 27.5
			
LVEF visits in database			
Mean (SD)	2.6 (1.1)	1.8 (1.1)	2.3 (1.1)
Quartiles (25th, median, 75th)	2.0, 3.0, 3.0	1.0, 1.0, 2.0	1.0, 2.0, 3.0
			
Time between first and last LVEF visit (months)			
Mean (SD)	18.1 (13.4)	11.0 (14.5)	15.2 (14.1)
Quartiles (25th, median, 75th)	9.0, 19.9, 27.9	0.0, 0.0, 23.7	0.0, 14.3, 23.7
			
Time between baseline and worst LVEF (months)			
Mean (SD)	26.2 (21.5)	20.2 (22.5)	23.7 (21.8)
Quartiles (25th, median, 75th)	8.0, 21.0, 39.3	1.6, 16.8, 27.5	7.2, 20.2, 30.3

Of the 19 subjects who had low BMD, 15 had more than one LVEF measurement compared to 6 subjects who had normal BMD. The mean change in LVEF measurement from the baseline to the lowest measurement was -1.9%. The mean decline in LVEF measurement from baseline to the lowest was not statistically significant between those with low BMD versus normal BMD (-1.8% and ‑2.0%, respectively).

**Bubble plot of worst LVEF vs. baseline BMD Z-score:**
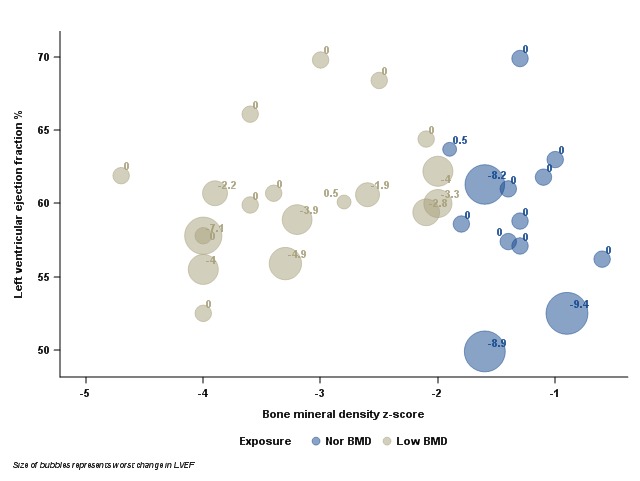

In the primary unadjusted model, bone mineral density did not explain any of the total variance in LVEF (*ß*=-0.00067, p‑value=0.9993). Age, months between baseline bone mineral density and worst LVEF, and steroid use were included in the adjusted models as clinically meaningful confounders. After controlling for age, months between baseline bone mineral density and worst LVEF, and steroid use, the full model explained 14.6% of the total variance in LVEF.

***Sensitivity analyses***

In the first planned secondary analysis, linear regression was calculated with LVEF as the dependent variable measured at approximately 24 months following baseline DEXA, with BMD as the independent variable. In the unadjusted model, BMD explained 1.1% of the total variance in LVEF (ß=‑0.64, p‑value=0.7879). After controlling for age and steroid use, the full model explained 9.6% of the total variance in LVEF (ß=-2.12, p-value=0.5984).

In the second planned secondary analysis, linear regression was calculated with the lowest LVEF as the dependent variable and BMD as the independent variable, excluding the four patients who had an imputed baseline BMD Z-score. In the unadjusted model, BMD explained 5.5% of the total variance in LVEF (ß=-1.02, p‑value=0.2282). After controlling for age, time in months between baseline BMD and lowest LVEF, and steroid use, the full model explained 17.6% of the total variance in LVEF (ß=-1.45, p-value=0.2382).

Finally, in the third planned secondary analysis, linear regression was calculated with change in LVEF as the dependent variable and BMD as the independent variable. In the unadjusted model, BMD explained 1.8% of the total variance in change in LVEF (ß=-0.38, p‑value=0.4919). After controlling for age, time in months between baseline BMD and lowest LVEF, and steroid use, the full model explained 29.6% of the total variance in LVEF (ß=-0.55, p‑value=0.1564). Table 2

**Table 2. Linear regression results of the sensitivity analyses:** Age, months from baseline BMD to worst LVEF, and steroid use included in the models as covariates.

Baseline BMD	ß	R^2^	p-value
Analysis 1 (only patients with LVEF visit ~24 months from baseline BMD)	-2.12	0.10	0.598
Analysis 2 (no imputation)	-1.45	0.18	0.238
Analysis 3 (change in LVEF)	-0.55	0.30	0.156

## Discussion

In this study, we explored the relationship between BMD and cardiovascular health, and observed no association between BMD and LVEF. The primary results were in the opposite direction than the hypothesized direction, with the model beta coefficient for BMD indicating a negative relationship with LVEF; however, the size of the effect BMD had on LVEF was very small and not statistically significant. The secondary linear regression analyses provided consistent results with the primary linear regression, indicating that the primary results are sensitive to a variety of slightly modified conditions.

It is possible that the null finding between bone mineral density and LVEF may be partially explained by a higher proportion of the patients with low BMD receiving steroids. There is unequivocal evidence that glucocorticoids alter the natural history of DMD.^9^ In this particular dataset, there was not sufficient evidence to detect a statistically significant relationship between steroid use and LVEF; similarly, the association between BMD and LVEF remained null after controlling for steroid use.

The strongest association reported, with the model explaining approximately 30% of the variance in LVEF, came from the third adjusted secondary analysis looking at change in LVEF as the outcome. Although the results were not statistically significant for BMD, this adjusted analysis reported the smallest p-value for BMD (0.1564) and the model F-statistic was statistically significant (p-value=0.0432). This data suggests that serial assessment of LVEF in DMD may not only inform cardiac health, but may also have added advantage of assessing the additional risk from having low BMD. Our findings also highlight the importance of carefully interpreting LVEF; the change in LVEF over time, and how quickly LVEF changes may be as informative and clinically meaningful of cardiac health, as the actual LVEF measurement.

Our study has some limitations. The small sample size and the retrospective study design do not permit a broad generalizability of the risk of low BMD and cardiovascular events in this population. In line with following cardiac screening as part of standard-of-care,^10^,^11^ we did find that those with lower BMD were older and had more LVEF measurements compared to the boys with normal BMD. Most subjects did not show a large change in LVEF during the study period, suggesting that a longer, prospective study is needed to evaluate the true risk between BMD and cardiovascular risk in DMD. Despite the limitations, this present study, informs us of the potential overlap in pathophysiological mechanisms between bone health and cardiovascular health. Future studies in DMD should consider longitudinal and timed evaluation of both bone health and cardiovascular health.

## Data availability

The minimal anonymized dataset is freely available at: https://figshare.com/articles/Duchenne_BMD_LVEF_xls/6004871. Questions regarding the permissions for the collection and sharing of this dataset should be directed to Naomi Luban, the Chair of the Children's National Health System IRB (nluban@childrensnational.org)

## Competing Interests

The authors have declared no competing interests exist.

## Corresponding author

Tara Anne Kervin, M.P.H., taraannekervin@gmail.com
